# Using tumor habitat-derived radiomic analysis during pretreatment ^18^F-FDG PET for predicting KRAS/NRAS/BRAF mutations in colorectal cancer

**DOI:** 10.1186/s40644-024-00670-2

**Published:** 2024-02-12

**Authors:** Hongyue Zhao, Yexin Su, Yan Wang, Zhehao Lyu, Peng Xu, Wenchao Gu, Lin Tian, Peng Fu

**Affiliations:** 1 Department of Nuclear Medicine, The First Affiliated Hospital of Harbin Medical University, Harbin, Heilongjiang China; 2 Department of Pathology, The First Affiliated Hospital of Harbin Medical University, Harbin, Heilongjiang China; 3 Department of Diagnostic and Interventional Radiology, University of Tsukuba, Ibaraki, Japan

**Keywords:** Colorectal cancer, KRAS/NRAS/BRAF, Habitat, Radiomic, ^18^F-FDG PET

## Abstract

**Background:**

To investigate the association between Kirsten rat sarcoma viral oncogene homolog (KRAS) / neuroblastoma rat sarcoma viral oncogene homolog (NRAS) /v-raf murine sarcoma viral oncogene homolog B (BRAF) mutations and the tumor habitat-derived radiomic features obtained during pretreatment ^18^F-fluorodeoxyglucose (FDG) positron emission tomography (PET) in patients with colorectal cancer (CRC).

**Methods:**

We retrospectively enrolled 62 patients with CRC who had undergone ^18^F-FDG PET/computed tomography from January 2017 to July 2022 before the initiation of therapy. The patients were randomly split into training and validation cohorts with a ratio of 6:4. The whole tumor region radiomic features, habitat-derived radiomic features, and metabolic parameters were extracted from ^18^F-FDG PET images. After reducing the feature dimension and selecting meaningful features, we constructed a hierarchical model of KRAS/NRAS/BRAF mutations by using the support vector machine. The convergence of the model was evaluated by using learning curve, and its performance was assessed based on the area under the receiver operating characteristic curve (AUC), calibration curve, and decision curve analysis. The SHapley Additive exPlanation was used to interpret the contributions of various features to predictions of the model.

**Results:**

The model constructed by using habitat-derived radiomic features had adequate predictive power with respect to KRAS/NRAS/BRAF mutations, with an AUC of 0.759 (95% CI: 0.585–0.909) on the training cohort and that of 0.701 (95% CI: 0.468–0.916) on the validation cohort. The model exhibited good convergence, suitable calibration, and clinical application value. The results of the SHapley Additive explanation showed that the peritumoral habitat and a high_metabolism habitat had the greatest impact on predictions of the model. No meaningful whole tumor region radiomic features or metabolic parameters were retained during feature selection.

**Conclusion:**

The habitat-derived radiomic features were found to be helpful in stratifying the status of KRAS/NRAS/BRAF in CRC patients. The approach proposed here has significant implications for adjuvant treatment decisions in patients with CRC, and needs to be further validated on a larger prospective cohort.

**Supplementary Information:**

The online version contains supplementary material available at 10.1186/s40644-024-00670-2.

## Introduction

Around 800,000 people across the world succumb to colorectal cancer (CRC) every year. It is the third most common type of cancer, after lung and breast cancers [1]. Epidermal growth factor receptor (EGFR) signaling is a frequent event in cancer development [2]. The gene-encoding downstream effectors of the EGFR pathway in CRC include Kirsten rat sarcoma viral oncogene homolog (KRAS), neuroblastoma rat sarcoma viral oncogene homolog (NRAS) and v-raf murine sarcoma viral oncogene homolog B (BRAF) [3]. The KRAS and NRAS genes are both part of the rat sarcoma viral oncogene (Ras) family of small GTPases. KRAS mutations occur in the early stage of CRC, with an incidence of 30%–50%, while NRAS has an incidence of 3% of all cases [4, 5]. The BRAF gene, which belongs to the serine/threonine kinase family, is a proto-oncogene, and mutations in it occur in about 10% of CRC patients [4, 5]. The activation of oncogenic mutations in KRAS/NRAS/BRAF can result in constitutive Ras/Raf/MEK/ERK activation, where are key drivers of cancer growth in humans and indicate poor survival poor survival [6, 7]. In other words, mutations in Ras genes (such as KRAS and NRAS) or BRAF genes can activate downstream signaling pathways without EGFR inhibition, leading to tumor proliferation [8]. Accordingly, the National Comprehensive Cancer Network recommends that the KRAS/NRAS/BRAF genotypes in metastatic CRC patients be determined [9], and has approved both panitumumab and cetuximab as anti-EGFR monoclonal antibodies for the treatment of metastatic CRC caused by KRAS/NRAS/BRAF wild-type tumors [10, 11].

Previous studies have explored the mechanisms underlying the relationship between glucose accumulation and the status of KRAS/NRAS/BRAF mutation in CRC cells [12]. However, studies on the predictive value of the metabolic parameters have yielded different conclusions [4, 13]. The accumulation of ^18^F-fluorodeoxyglucose (FDG) in cancer tissues is a complex process that is influenced by various mechanisms, such as mutated TP53, HIF-1α expression, and local inflammation, that can interfere with ^18^F-FDG uptake by CRC cells [12, 14, 15]. Therefore, simple metabolic parameters struggle to identify the metabolic heterogeneity in different regions within the tumor. Moreover, the values of the most commonly used metabolic parameters in positron emission tomography (PET) vary with different factors, including the type of PET scanner used, metabolic differences between patients, and their blood glucose levels after fasting [4]. The recent emergence of radiomics technology, which can be used to extract microscale imaging-related information from standard medical images to predict complex genomic events, provides an alternative mechanism [16]. Nevertheless, a retrospective study of 151 patients with rectal cancer showed that textural features based on ^18^F-FDG PET/computed tomography (CT) have limited value in terms of predicting KRAS/NRAS/BRAF mutations [17]. Furthermore, the use of radiomics based on magnetic resonance to assess KRAS mutations does not always yield accurate results [18, 19]. Most radiomics studies usually analyze the tumor as a whole. However, this approach assumes that the tumor is heterogeneous but well mixed, thus, ignoring local phenotypic differences within the tumor [20]. In contrast to previously proposed methods, partitioning groups of voxels with a similar tumor biology into subregions for analysis—namely, habitat imaging—allows for better quantification of heterogeneity within tumors [21, 22]. Habitat-derived radiomics analysis in particular provides a better insight into the tumor heterogeneity than traditional radiomics analysis, in terms of the prognosis and predictions of the survival of patients of esophageal cancer who have been treated with concurrent chemoradiotherapy [23]. Hence, we hypothesize that this technique can be used to predict KRAS/NRAS/BRAF mutations in CRC patients and contribute to the development of modalities of non-invasive genomic detection.

Although mutation analysis using tissues of the tumor remains the standard of care [24], overcoming the limitations of spatial and temporal heterogeneity in the tumor based on the information provided by imaging-based studies has profound implications for adjunctive personalized molecular therapy. In this pilot study, we aim to determine whether habitat-derived radiomics analysis is an effective means to distinguish between mutant genomes and wild-type genomes in CRC patients, and to compare habitat-derived radiomics analysis with whole tumor region radiomics analysis and the metabolic parameters.

## Materials and methods

### Patients

The experimental protocols were approved by the Institutional Ethics Committee of the First Affiliated Hospital of Harbin Medical University, and the requirement of written informed consent from the patients was waived owing to the retrospective nature of this study. We collected data on patients with CRC who had undergone ^18^F-FDG PET/CT imaging and surgical treatment in the hospital from January 2017 to July 2022, for a total of 245 cases. As part of the analysis, we gathered clinical information on the patients and their pathological characteristics from medical records, including age, sex, tumor location, tumor differentiation, TNM stage, carcinoembryonic antigen (CEA), and KRAS/NRAS/BRAF status. The mutation analysis of KRAS (exons 2, 3, and 4), NRAS (exons 2, 3, and 4), and BRAF (V600E) was performed by using the amplification refractory mutation system–PCR methods in formalin-fixed and paraffin-embedded tumor samples. The criteria of exclusion were as follows: (1) patients who had received antineoplastic therapy (such as surgery, radiotherapy, and chemotherapy) before PET/CT examination, (2) no PET/CT scan or incomplete clinical information, (3) the interval between PET/CT imaging and genetic testing was longer than four weeks, (4) negative ^18^F-FDG uptake or volume of interest (VOI) measuring was smaller than 64 voxels, and (5) patients with other malignant tumors. A total of 62 CRC patients were finally considered for the analysis. They consisted of 41 males and 21 females, with a mean age of 63.06 years (range, 32–83 years). The cases were then randomly included in either a training cohort (*n* = 37) or a validation cohort (*n* = 25) by using stratified sampling with a 6:4 ratio.

### PET/CT imaging

Each patient had undergone ^18^F-FDG PET/CT examination on a Gemini GXL PET/CT scanner (Philips, Amsterdam, Netherlands) that was equipped with a 16-slice CT. An HM-12 cyclotron (Sumitomo Heavy Industries Ltd., Tokyo, Japan) was used for the production of ^18^F-FDG with a high radiochemical purity (≥ 95%). The patients were made to fast for at least 6 h before the intravenous injection of ^18^F-FDG (3.7 ~ 7.4 MBq/kg), and their intravenous blood glucose was controlled to below 8.0 mmol/L. Images were acquired 60 ± 5 min after the injection of the ^18^F-FDG tracer. The patients were scanned from the middle of the thigh to the top of the skull according to the standard institutional clinical protocols. Whole-body scanning with a low-dose CT (tube current: 50 mA, tube voltage: 120 kV, slice thickness: 5 mm) was performed without an oral or an intravenous contrast agent, followed by PET imaging (1.5 min/bed position, six to seven PET bed positions, 50% overlap between bed positions) in the same range. The data were reconstructed using the 3D-line of response RAMLA reconstruction algorithm that corrected for attenuation, attenuation decay, scatter, and dead time. The PET/CT images were fused using Syntegra V2.1 J software (Philip Medical Systems, Netherlands). Each reconstructed PET image had a matrix size of 114 × 114 × 129, and a voxel size of 4 × 4 × 4 mm, while each reconstructed CT image had a matrix size of 512 × 512 × 211, and a voxel size of 1.172 × 1.172 × 5.000 mm.

### Tumor segmentation and habitat generation

**Fig. 1:**
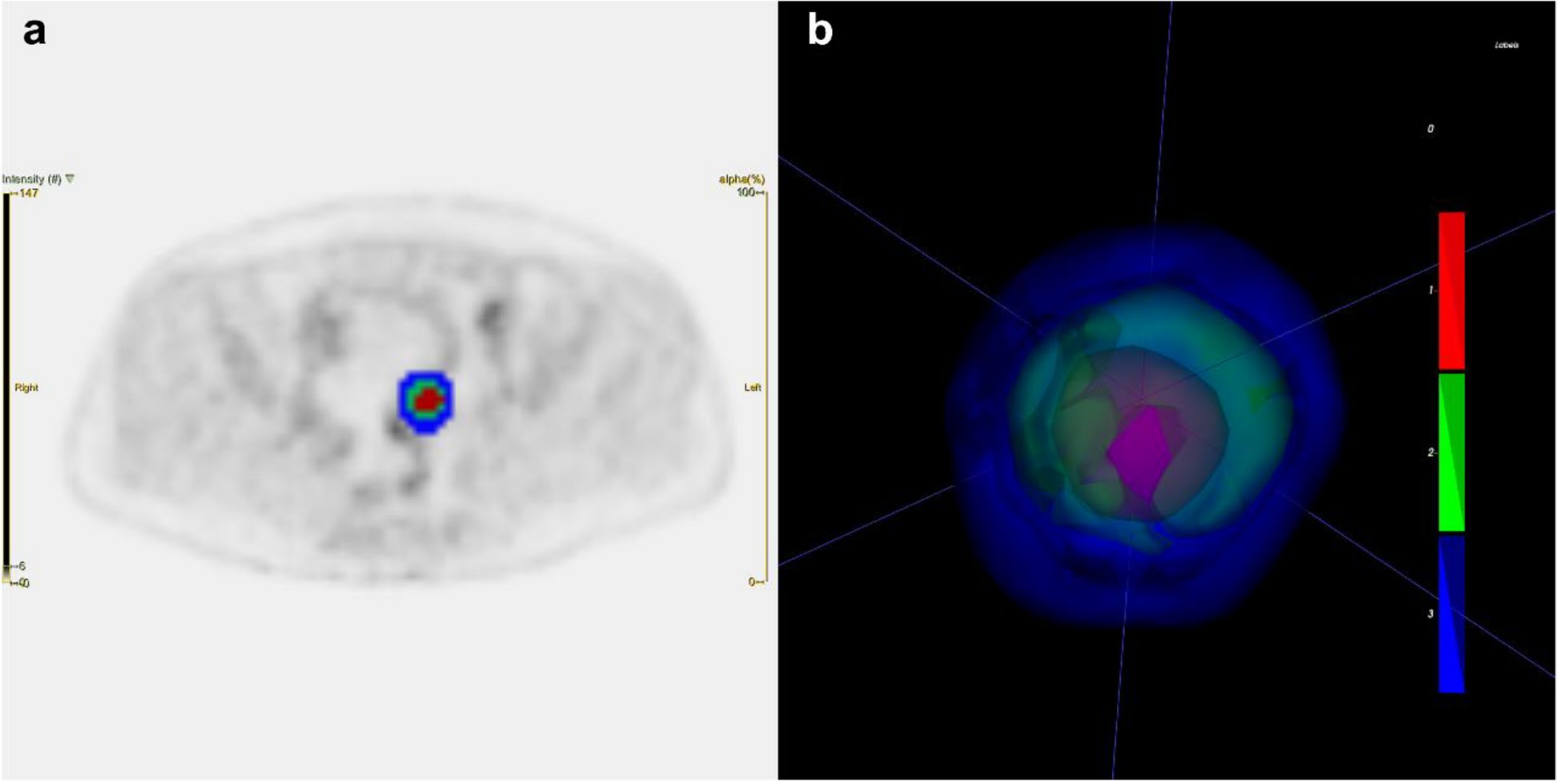
Schematic diagram of habitat generation. **a** Three subregions, low_metabolism_habitat (green area), hight_metabolism_habitat (red area) and peritumoral (blue area) were obtained from the PET images. **b** A three-dimensional volume-rendered image of the three subregions. PET, positron emission tomography

The acquired PET/CT images were anonymized. The PET images were normalized to injection doses of ^18^F-FDG and the body weight of the patients after decay correction to transform them into standardized uptake value (SUV) units. All images were observed in a range of threshold of SUV from 0 to 6, and the two-dimensional region of interest was manually segmented based on the boundary of the tumor lesion on each horizontal axial PET image with tumor to form a three-dimensional VOI. The VOI of the tumor was segmented by a physician experienced in nuclear medicine (Z.L.; 8 years of experience), who had been unaware of the status of mutation, by using LIFEx software (version 7.2.0; www.lifexsoft.org) [25]. The Otsu threshold algorithm was implemented in Python (version 3.9.7; www.python.org). It can be used to count the number of pixels of each gray level in the VOI and identify an “optimal” threshold to maximize the inter-class variance [26, 27]. The corresponding VOI of the tumor in the PET images was divided into two significantly different hypermetabolic and hypometabolic regions representing different tumor habitats. We defined them as “low_metabolism_habitat” and “high_metabolism_habitat,” respectively. In addition, a ring-shaped peritumoral VOI was generated by expanding the margin of the lesion by 2 mm according to the outline of the segmented tumor, and special care was taken to avoid including other structures, especially the bladder and adjacent lymph nodes, by making additional modifications to the peritumoral VOI as needed. We explicitly considered the peritumoral tissue as a unique region of the tumor habitat to account for the relationship between mutations of genes of the tumor and the peritumoral regions. In this way, three subregions—low_metabolism_habitat, high_ metabolism_habitat, and peritumoral—were obtained from the PET images, as shown in Fig. 1.

### Feature extraction

The data on each raw image were resampled to an isotropic resolution of 1 × 1 × 1 mm^3^ by using the B-Spline interpolation method, and all VOIs defined in the raw imaging data were resampled by using the Nearest Neighbor interpolation on the data on isotropic resolution. The radiomic features were extracted by using PyRadiomics (version 3.0.1; https://github.com/Radiomics/pyradiomics), an open source radiomics toolkit [28]. A fixed bin width of 0.25 SUV was applied to discretize the continuous scale of SUV intensity and calculate the textural features [29, 30]. The extracted radiomic features (*n* = 107) were divided into seven categories: 14 shape features, 18 first-order statistics, 24 Gray-level co-occurrence matrix (glcm) features, 16 Gray-level size zone matrix (glszm) features, 16 Gray-level run length matrix (glrlm) features, 5 neighboring gray tone difference matrix (ngtdm) features, and 14 Gray-level dependence matrix (gldm) features. In addition, we applied a wavelet filter (wavelet-LHL, wavelet-LHH, wavelet-HLL, wavelet-LLH, wavelet-HLH, wavelet-HHH, wavelet-HHL, and wavelet-LLL) to the original images to obtain the corresponding derivative images in order to extract detailed high-dimensional radiomic features (*n* = 744) [31]. More interpretations are available from: https://www.radiomics.io/pyradiomics.html. A total of 851 features were thus calculated for each habitat, for a total of 2,553 (*n* = 851 × 3) radiomic features across the three habitats. We repeated the above procedure to extract 851 radiomic features from the entire region of the tumor in each patient for the sake of comparison.

To assess the robustness and stability of the obtained radiomic features, the nuclear medicine physician (Z.L.) and another experienced radiologist (W.G.; 8 years of experience) independently performed repeated segmentation on 20 randomly selected cases after a one-month interval. This was done to calculate the intra-observer and inter-observer correlation coefficients (ICCs). Higher values of the ICCs reflected a higher degree of reproducibility, and features with ICCs below 0.75 were excluded from subsequent analyses.

For a comparison of predictive performance, eight metabolic parameters—minimal standardized uptake value (SUVmin), average standardized uptake value (SUVmean), maximal standardized uptake value (SUVmax), standard deviation of standardized uptake value (SUVstd), metabolic tumor volume (MTV), standardized metabolic tumor volume (sMTV), total lesion glycolysis (TLG), standardized total lesion glycolysis (sTLG) were also calculated by using voxels larger than the set threshold of 40% of SUVmax in the VOI of the tumor. The detailed formula has been provided on the relevant website (www.lifexsoft.org).

### Correlation analysis of features

Prior to feature selection and model construction, we performed the unsupervised clustering of the habitat-derived radiomic features, the whole tumor region radiomic features, and the metabolic parameters by using the Euclidean distance. This allowed us to determine the differences between the patterns of global expression of features of the images in the presence and absence of KRAS/NRAS/BRAF mutations. Moreover, we visualized the correlation between the habitat-derived radiomic features and whole tumor region radiomic features, as well as that between the habitat-derived radiomic features and the metabolic parameters by using pairwise Pearson correlation coefficients to create correlation heatmaps. This enabled us to link different features of the images.

### Feature selection and prediction model development

To begin with, each feature was z-score normalized by using the mean and standard deviation of the training cohort features. The addition of a feature-ranking procedure beforehand can help improve the final performance [32]. We thus used the entropy-based maximum relevance minimum redundancy (mRMR) technique of feature selection in the supervised stage of feature selection. Entropy-based mRMR algorithms use mutual information and conditional entropy to measure the correlation and redundancy between features. Specifically, the algorithm first calculates the mutual information between each feature and the target variable, and then calculates the conditional entropy between the selected feature and other features, to select the optimal feature that has the maximum correlation with the target variable and the minimum redundancy with other features. The top 30 radiomic features were then fed into the least absolute shrinkage and selection operator (LASSO) algorithm for more refined feature selection. The optimal coefficient of lambda shrinkage within one standard error of this value was determined using ten-fold cross-validation. By penalizing the loss function with the L1 norm of the feature coefficients, LASSO forced those coefficients corresponding to weak features to become zero, thus preventing overfitting to produce a sparse matrix [33]. The underlying relationship between the radiomic phenotypic features and the genetic status is complex, and may even be non-linear. Moreover, the sample size in this study was small. Therefore, we used the support vector machine (SVM) to build a model of the classifier. The SVM is a statistical classifier model based on the principle of structural risk minimization that has been used to solve a range of non-linear problems involving a small number of samples [34, 35]. Five-fold cross-validation and random search with 1,000 iterations were used to determine the best parameter configuration during model training. The numbers of iterations and folds of cross-validation were determined based on factors such as the trade-off of bias and variance, as well as the actual computational resources. The same workflow was used for the habitat-derived radiomic features and the whole tumor region radiomic features. However, owing to the small number of dimensions of their features, we used univariate analysis to input features of the metabolic parameters that were significantly different into the SVM classifier, with a value of P smaller than 0.05 as the threshold.

### Model evaluation and validation

The predictive performance of the model was evaluated on the entire training cohort and validated by using the validation cohort. We applied the receiver operating characteristic (ROC) curve, calibration curve, and decision curve analysis (DCA) to evaluate the predictive accuracy and clinical feasibility of the model. We also calculated the area under the receiver operating characteristic curve (AUC), sensitivity, specificity, accuracy, recall, precision, the F1-score, and area under the precision–recall curve (AUPR) as indicators of performance. The learning curve of the model was used to determine its convergence. It is a visualization tool to identify whether the number of training samples of a given model is sufficient for its convergence by showing how the errors or accuracies of the training and validation sets change as the number of training samples increases [36]. We used the SHapley Additive explanation (SHAP) method, which is a visual tool that can calculate the contribution of each input variable to the decision of a machine learning model [37], to interpret the output of the machine learning model, and reveal the relationship between the radiomic features of the image and KRAS/NRAS/BRAF mutations.

### Statistical analysis

The Python programming language (version 3.9.7; www.python.org) was used for statistical analysis, visualization, and machine learning. The Student t-test was used to determine whether the continuous variables were normally distributed (the results are reported as mean ± standard deviation), and the Mann–Whitney U test was used if they were not normally distributed (presented as the median with an interquartile range). The Chi-square test or Fisher’s exact test was used for the categorical variables (presented as numbers or percentages). Two-tailed *P*-values of < 0.05 were considered to be statistically significant.

## Results

### Clinical characteristics

The patients were divided into a mutant group and a wild group according to the results of the status of KRAS/NRAS/BRAF mutations. Table 1 lists the demographics of the patients and the clinical characteristics of the tumors in both groups. No statistical differences were observed between the wild and mutant groups in terms of sex, age, tumor location, and differentiation (*P* > 0.05). However, statistically significant differences were noted between the wild and mutant groups in terms of the TNM stage and the CEA (*P* < 0.05). Table 2 lists the demographics of the patients and the clinical characteristics of the tumor in the training and validation groups. The results show that although there were differences between them in terms of sex, age, tumor location, TNM stage, and CEA, they were not statistically significant (*P* > 0.05).

**Table 1:** Comparison of patient demographic and clinical tumor characteristics between KRAS/NRAS/BRAF mutation group and wild type group

Characteristics	Wild type (*n* = 27)	Mutant type (*n* = 35)	P
Sex			0.644^a^
Male	17(63.0)	24(68.6)	
Female	10(37.0)	11(31.4)	
Age(year)	61.11 ± 11.40	64.57 ± 10.21	0.213^b^
Tumor location			0.636^a^
Colon or sigmoid colon	20(74.1)	24(68.6)	
Rectum or rectosigmoid junction	7(25.9)	11(31.4)	
Differentiation			0.420^a^
Well/moderate	21(77.8)	24(68.6)	
Poor	6(22.2)	11(31.4)	
TNM stage			0.044^a^
I-II	17(63.0)	13(37.1)	
III-IV	10(37.0)	22(62.9)	
CEA			0.001^a^
< 5.0 ng/ml	21(77.8)	12(34.3)	
≥ 5.0 ng/ml	6(22.2)	23(65.7)	

*CEA* carcinoembryonic antigen, *KRAS* Kirsten rat sarcoma viral oncogene homolog, *NRAS* neuroblastoma rat sarcoma viral oncogene homolog, *BRAF* v-raf murine sarcoma viral oncogene homolog B

^a^Chi-square test

^b^Student t-tests

**Table 2:** Comparison of patient demographic and clinical tumor characteristics between the training cohort and validation cohort

Characteristics	Training cohort (*n* = 37)	Validation cohort (*n* = 25)	P
Sex			0.402^a^
Male	26(70.3)	15(60.0)	
Female	11(29.7)	10(40.0)	
Age(year)	64.35 ± 10.71	61.16 ± 10.85	0.257^b^
Tumor location			0.473^a^
Colon or sigmoid colon	25(67.6)	19(76.0)	
Rectum or rectosigmoid junction	12(32.4)	6(24.0)	
Differentiation			0.933^a^
Well/moderate	27(73.0)	18(72.0)	
Poor	10(27.0)	7(28.0)	
TNM stage			0.640^a^
I-II	17(45.9)	13(52.0)	
III-IV	20(54.1)	12(48.0)	
CEA			0.498^a^
< 5.0 ng/ml	21(56.8)	12(48.0)	
≥ 5.0 ng/ml	16(43.2)	13(52.0)	
Mutational status			0.953^a^
KRAS/NRAS/BRAF mutated	21(56.8)	14(56.0)	
Wild type	16(43.2)	11(44.0)	

*CEA* carcinoembryonic antigen, *KRAS* Kirsten rat sarcoma viral oncogene homolog, *NRAS* neuroblastoma rat sarcoma viral oncogene homolog, *BRAF* v-raf murine sarcoma viral oncogene homolog B

^a^Chi-square test

^b^Student t-tests

### Unsupervised cluster analysis and correlation analysis

After excluding features with low reproducibility (ICCs < 0.75), the number of habitat-derived radiomic features were reduced from 2553 to 2426, and the number of whole tumor region radiomic features were reduced from 851 to 803. We then separately analyzed their clustering. The cluster map of the habitat-derived radiomic features had a clear contrast in shape with the cluster maps of the whole tumor region radiomic features and the metabolic parameters (Supplementary Information Fig. S1). This indicates that the habitat-derived radiomic features were more representative and compact. Notably, the heatmap of correlation (Supplementary Information Fig. S2) showed that habitat-driven radiomics features with potential relevance could be found for almost all whole tumor region radiomic features or metabolic parameters (dark-blue color). However, the presence of partial habitat-derived radiomic features exhibited a low correlation with the whole tumor region radiomic features or the metabolic parameters (light-blue and red colors, respectively). This may correspond to additional deep information and be more suggestive for the status of KRAS/NRAS/BRAF mutations during model construction.

### Feature selection

**Fig. 2:**
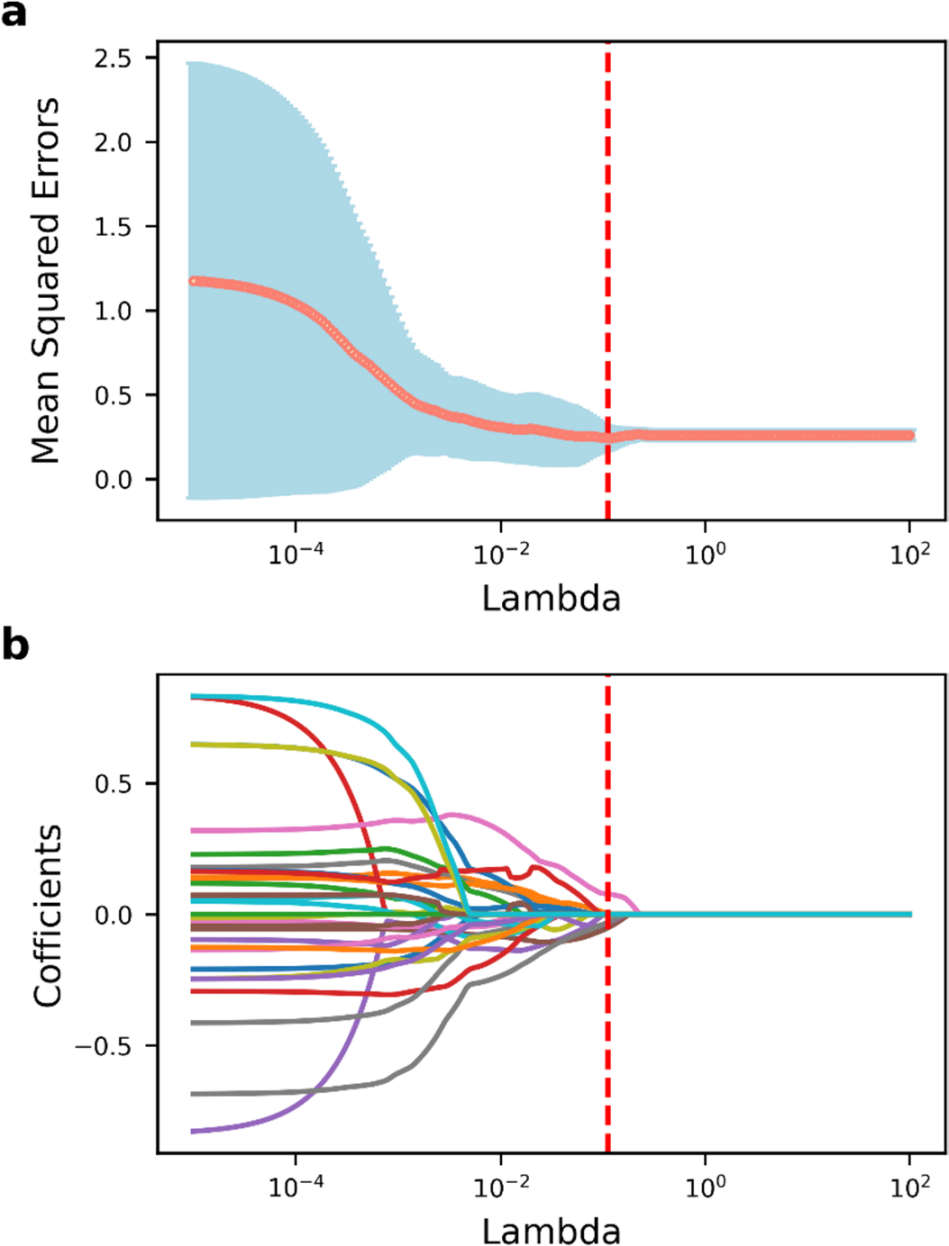
Feature selection of habitat-derived radiomic features using the LASSO algorithm. **a** Tuning parameter lambda selection in the LASSO algorithm used ten-fold cross-validation. **b** LASSO coefficient profiles of the features. LASSO, least absolute shrinkage and selection operator

The habitat-derived radiomic features were ranked according to mRMR, and the top 30 features were then selected for further analysis. The top features included seven features from low_metabolism_habitat, 12 from high_metabolism_habitat, and 11 from the peritumoral region. Following this, we used the LASSO method to select five subregional radiomic features with non-zero coefficients under the best value of lambda, as illustrated in Fig. 2. We finally retained two features from high_metabolism_habitat, three from the peritumoral region, and no feature from low_metabolism_habitat. The rule of thumb to avoid overfitting is that the number of predictors should be kept within 1/10–1/3 of the sample size in each group of the main cohort [38]. Therefore, the number of features screened here was considered reasonable. However, when mRMR and LASSO analyses were performed on the whole tumor region radiomic features, no feature was retained (Supplementary Information Fig. S3). We also did not find a significant difference in the metabolic parameters between the wild type group and the mutant group of KRAS/NRAS/BRAF, although all metabolic parameters exhibited a tendency to have larger values in the mutant group than in the wild type group (Supplementary Information Fig. S4). Therefore, no whole tumor region radiomic features and metabolic parameters were available for the subsequent examination of model construction.

### Model evaluation

**Fig. 3:**
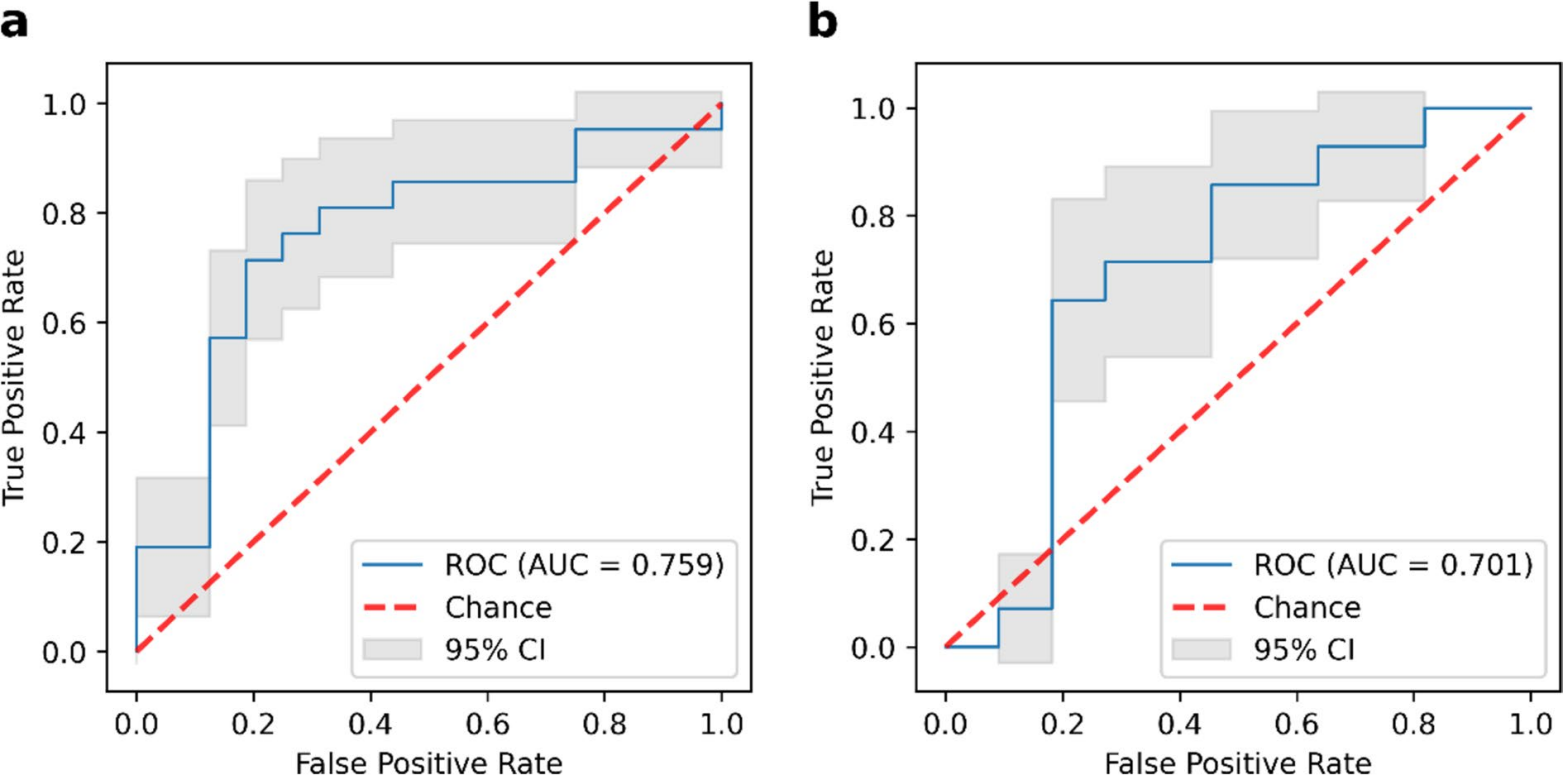
The ROC curves of the SVM model based on habitat-derived radiomic features in the training group (**a**) and the validation group (**b**). ROC, receiver operating characteristic; SVM, support vector machine; AUC, area under the receiver operating characteristic curve

**Fig. 4:**
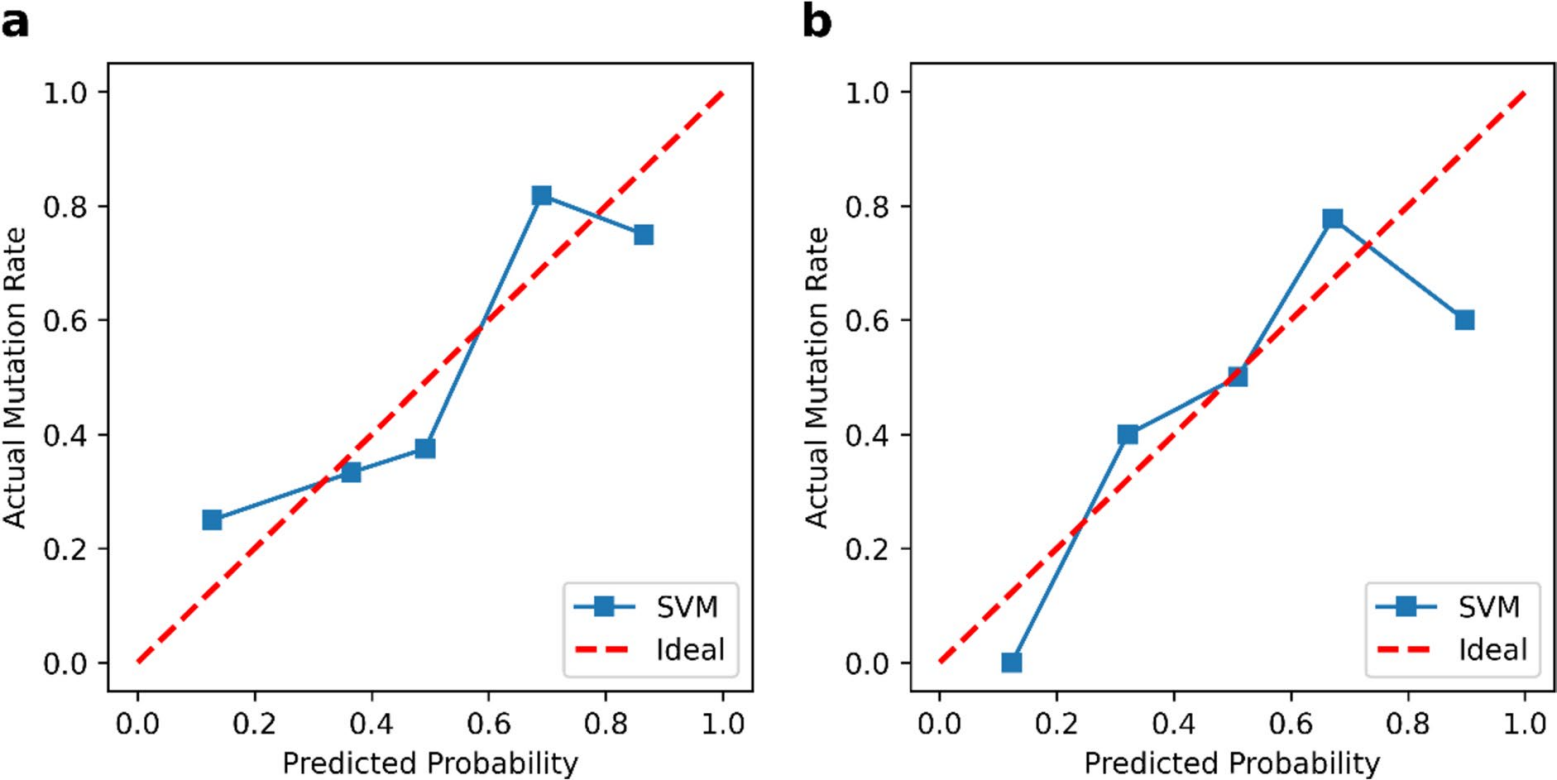
The calibration curves of the SVM model based on habitat-derived radiomic features in the training group (**a**) and the validation group (**b**). SVM, support vector machine

**Fig. 5:**
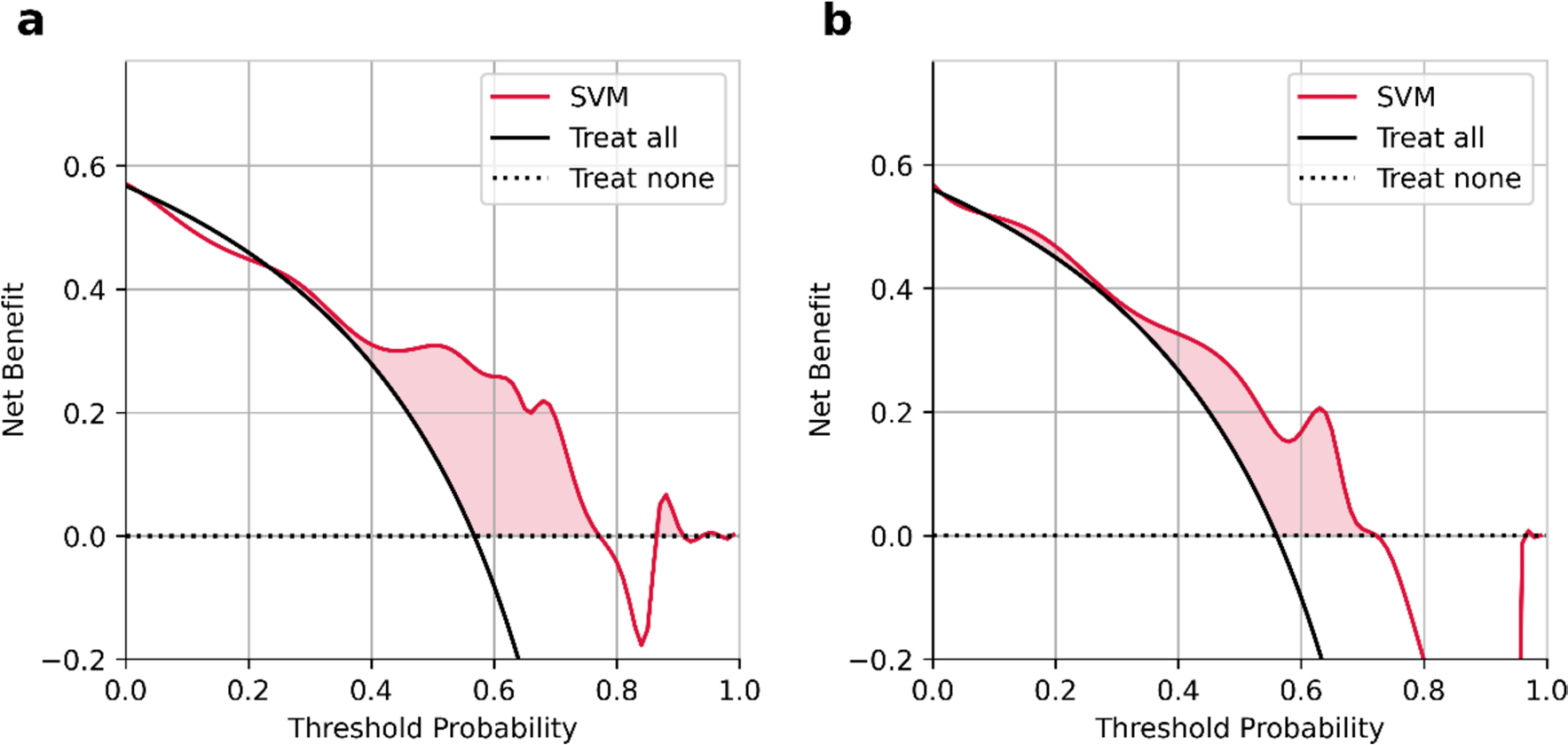
The DCA of the SVM model based on habitat-derived radiomic features for the training (**a**), and validation (**b**) groups. SVM, support vector machine; DCA, decision curve analysis

We constructed an SVM model to predict KRAS/NRAS/BRAF mutations based on the selected habitat-derived radiomic features, and determined the optimal values of the parameters as C = 2.815, gamma = 0.364, and kernel = sigmoid. The predictive performance of the model was subsequently tested on the training and validation sets. Table 3 and Fig. 3 show the performance of the model of classification and its ROC curves. The model had an AUC of 0.759 (95% CI: 0.585–0.909) on the training set, with a sensitivity of 0.810, specificity of 0.688, accuracy of 0.757, recall of 0.810, precision of 0.773, F1-score of 0.791, and AUPR of 0.808. The model in the validation group had an AUC of 0.701 (95% CI: 0.468–0.916), sensitivity of 0.786, specificity of 0.545, accuracy of 0.680, recall of 0.786, precision of 0.688, F1-score of 0.733, and AUPR of 0.682. These results reflect the acceptable predictive performance of the proposed model. In addition, its curve of calibration in Fig. 4 shows good agreement between the probability of KRAS/NRAS/BRAF mutations predicted by the model and their actual probability on both the training and validation datasets. Furthermore, the DCA plots verified the clinical utility of the SVM model based on habitat-derived radiomic features for the training group (Fig. 5a) and the validation group (Fig. 5b).

**Table 3:** Performance evaluation of SVM model based on habitat-derived radiomic features in training cohort and validation cohort. SVM, support vector machine; AUC, area under the receiver operating characteristic curve; AUPR, area under the precision‐recall curve

Cohort	AUC (95%CI)	Sensitivity	Specificity	Accuracy	Recall	Precision	F1-score	AUPR
Training cohort	0.759 (0.585–0.909)	0.810	0.688	0.757	0.810	0.773	0.791	0.808
Validation cohort	0.701 (0.468–0.916)	0.786	0.545	0.680	0.786	0.688	0.733	0.682

**Fig. 6:**
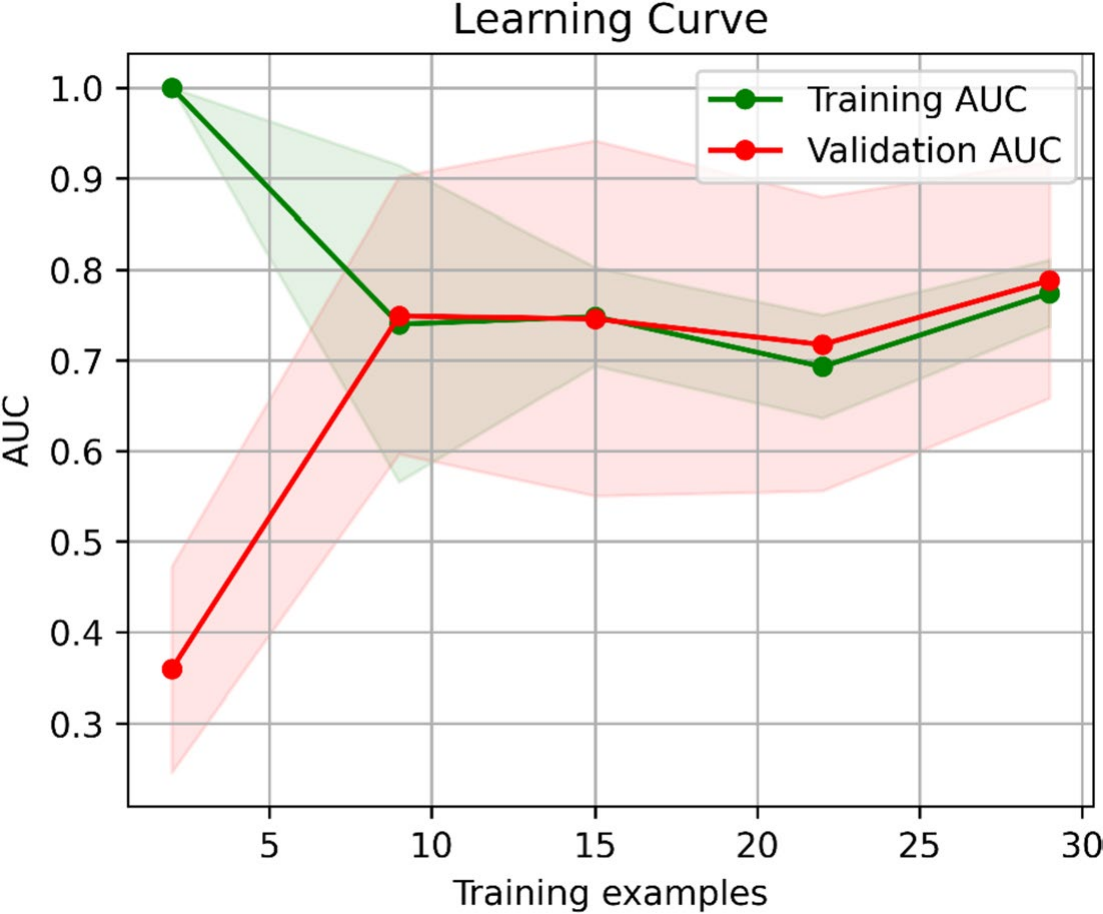
Learning curve of the SVM model based on habitat-derived radiomic features. SVM, support vector machine; AUC, area under the receiver operating characteristic curve

The learning curve provides a visual insight into the predictive performance of a model. The learning curve in Fig. 6 shows that values of the AUC of the proposed model on the training and validation groups tended to be stable and close to each other, and conformed to our expectations. Therefore, the proposed model converged and achieved stable performance even with a small sample size.

### Analysis of model interpretability

**Fig. 7:**
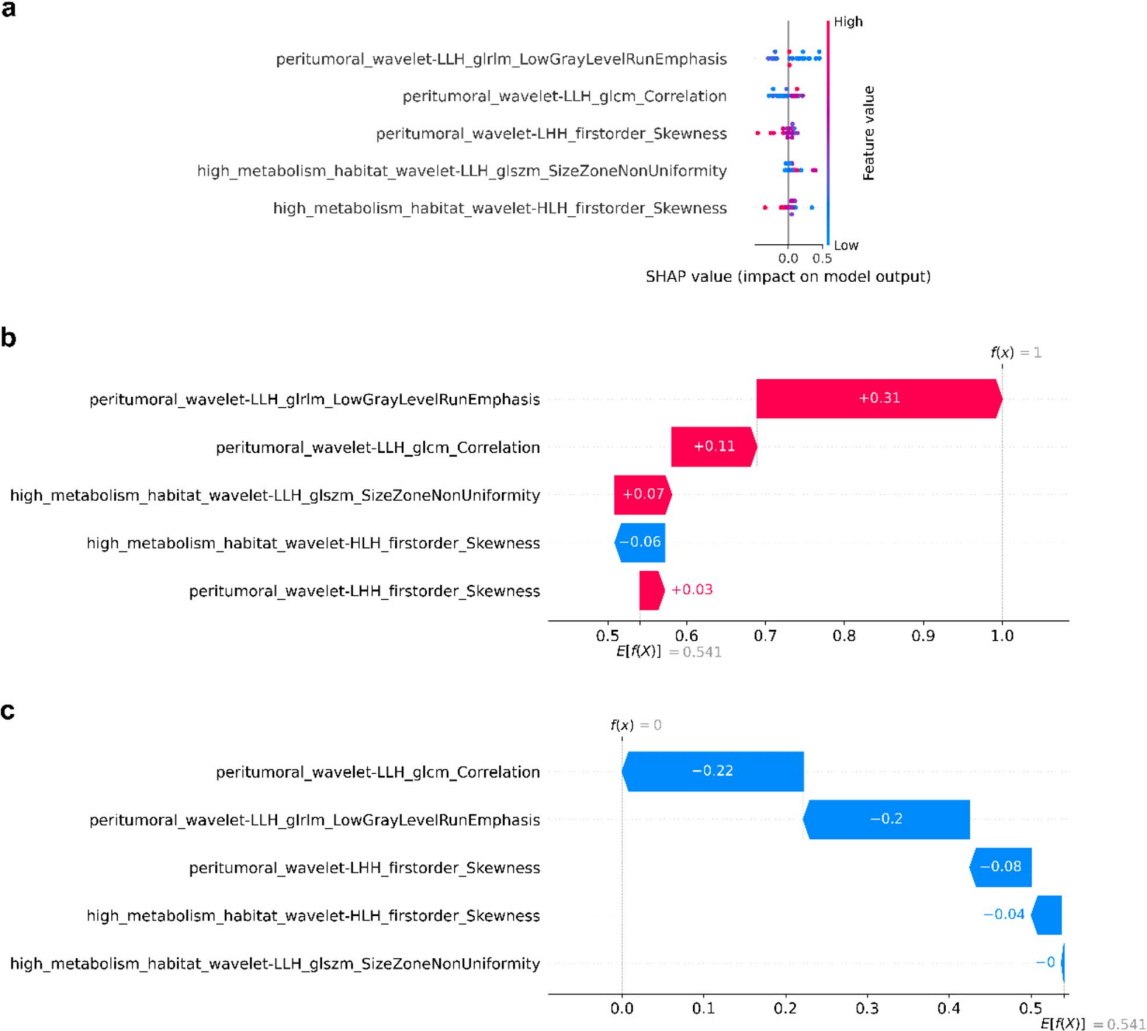
**a** SHAP summary graph and distribution of SHAP values for trained SVM model based on habitat-derived radiomic features. Characteristic SHAP value influence diagram of individual KRAS/NRAS/BRAF mutant (**b**) and wild type (**c**) samples. SHAP, SHapley Additive explanation; SVM, support vector machine; KRAS, Kirsten rat sarcoma viral oncogene homolog; NRAS, neuroblastoma rat sarcoma viral oncogene homolog; BRAF, v-raf murine sarcoma viral oncogene homolog B; glrlm, gray-level run length matrix; glcm, gray-level co-occurrence matrix; glszm, gray-level size zone matrix

SHAP analysis was conducted on the validation cohort. We calculated the importance of each feature based on the SHAP values (Fig. 7a). The graph shows the results of the analysis of the importance of features in the SVM model by SHAP method. Each point in Fig. 7a represents the SHAP value of a feature in one instance. Its position on the Y-axis was determined based on the importance of the feature and its position on the X-axis was determined by its SHAP value. The colors indicate values of the features ranging from small to large to better represent the distribution of the SHAP values for each feature. We also used SHAP to account for a typical prediction of the presence and absence of KRAS/NRAS/BRAF mutations (Fig. 7b and c). The predictions were made starting with a baseline value derived from the average of all predictions. Each band showed the effect of its feature in pushing the value of the target variable further from or closer to the baseline value, and the likelihood of increasing (positive value) or decreasing (negative value) KRAS/NRAS/BRAF mutations.

## Discussion

With advances in precision medicine, the status of KRAS/NRAS/BRAF mutations has been applied to clinical decision-making in case of CRC [39]. Histopathology remains the gold standard for the diagnosis and classification of tumors. However, there is increasing recognition of the limitations of tissue-based genetic testing, including intra-tumor heterogeneity, clonal evolution, and poor DNA quality, especially in biopsy samples [40]. This can lead to a suboptimal profile of the genetic signature of the tumor. Therefore, we sought to determine the most effective way to image phenotypes by using habitat-derived quantitative radiomic measurements to distinguish between CRC mutants and wild type genomes in this pilot study. The AUC of our SVM model was 0.759 on the training group and 0.701 on the validation group. It exhibited sound calibration and clinical utility, and achieved good convergence even with a small sample size. This suggests that it can complement the status of mutation of genes to enhance the optimal treatment strategy for patients with CRC.

Data from basic studies suggest that mutated KRAS may lead to increased ^18^F-FDG accumulation through the upregulation of GLUT1 under normoxic conditions, and that HIF-1α increases ^18^F-FDG accumulation in hypoxic lesions [12]. Therefore, it is theoretically feasible to characterize the phenotype of the mutants of tumors by using metabolic parameters. However, like previous studies [13], the results of ours showed that although the metabolic parameters of KRAS/NRAS/BRAF mutants tended to have larger values than in wild type genomes, none of these was statistical significance. Several parameters based on SUV (SUVmin, SUVmean, SUVmax, SUVstd) reflect the metabolic activity of a certain part or a certain point of the tumor tissue, but cannot reflect the overall metabolism of the tumor. The derived parameters (MTV, sMTV, TLG, sTLG) make up for this deficiency. For example, MTV and TLG can reflect the metabolic activity and the tumor burden. However, these parameters cannot distinguish the heterogeneity of different regions within the tumor [41]. We also observed that all whole tumor region radiomic features were removed during feature selection. Some previous studies have reported the same results, whereby whole tumor region textural features obtained by using ^18^F-FDG PET/CT are not associated with the presence of Ras mutations in patients of rectal cancer [17]. Fortunately, our SVM model based on habitat-derived radiomic features yielded good performance in predicting the presence of KRAS/NRAS/BRAF mutations, where this further demonstrates the value of techniques of habitat analysis in characterizing the complex microenvironment of tumors. It is important to note that different regions within the tumor may have different biological characteristics. Habitat-based methods deliver better performance, possibly due to the clustering of similar voxels through habitat quantification, which allows for the delineation of metabolic heterogeneity driven by genetic heterogeneity through a more fine-grained characterization of the microenvironment of the tumor [42]. The advantage of this promising approach to describing tumor heterogeneity has been demonstrated in several previous studies. For example, in their comparison of the performance of ^18^F-FDG PET/CT whole tumor region radiomics with habitat-derived radiomics in determining Ki-67 expression in case of high-grade serous ovarian cancer, Wang et al. found that the habitat-based method was significantly better than the whole tumor region radiomics analysis (Delong test, *P* < 0.05) [26]. In another study, Xie et al. used habitat-derived radiomic features in CT images to construct a model to assess the prognosis of esophageal squamous cell carcinoma treated by definitive concurrent chemoradiotherapy. The concordance index of the model was 0.729 on the training cohort and 0.705 on the validation cohort. This is superior to that of the whole tumor region radiomics analysis, although it was not statistically significant [23].

We also explored reasons for the poor performance of the metabolic parameters and the whole tumor region radiomics in comparison with that of habitat-derived radiomics by using unsupervised clustering analysis. The results showed that the unsupervised clustering of habitat-derived radiomics led to a better match with the semantic labels (mutant/wild type genomes). Moreover, the analysis of correlations between features showed that almost all whole tumor region radiomics features and metabolic parameters could find the corresponding habitat-derived radiomic features with correlation, and some habitat-derived radiomic features had a low correlation with the whole tumor region radiomic features and the metabolic parameters. Considering that our approach based on habitat-derived radiomics delivered better performance, it is reasonable to conclude that the relevant features not only cover almost all information on the metabolic parameters and the whole tumor region radiomic features, but also contain key discriminating information that is not found in these two sets of features of images. This also nicely explains the higher classification-related performance of habitat-derived radiomics.

We used SHAP analysis to interpret the “black-box model” at both the global and the local levels [43]. The SHAP method enhances the interpretability of a machine learning model, and can estimate the positive and negative contributions of each feature to its prediction [44]. The SHAP results showed that the ranking of the features according to importance was peritumoral_wavelet-LLH_glrlm_LowGrayLevelRunEmphasis, peritumoral_wavelet-LLH_glcm_Correlation, peritumoral_wavelet-LHH_firstorder_Skewness, high_metabolism_wavelet-LLH_glszm_SizeZoneNonUniformity, and high_metabolism_wavelet-HLH_firstorder_Skewness. The radiomic features obtained from the peritumoral regions were the three most important features, followed by those from the hypermetabolic regions, while none of the features from the hypometabolic regions was included in the final model. Tumor invasion is a complex biological process at the microscopic level, in which tumor cells tend to separate from the primary tumor and invade the surrounding tissues [45]. Tumors with gene mutations in particular are likely to invade and exhibit metastasized phenotypes [46]. Therefore, it may be beneficial to capture information on KRAS/NRAS/BRAF mutations from a macroscopic perspective by using radiomics in the peritumoral region. Multiple circular peritumoral regions were not observed in this study. According to a recent report, the closer the peritumoral area is to the intra-tumoral area, the greater is the amount of information that it contains [47]. The excessive expansion of information on the surrounding edges may introduce noise to impair the capability of classification of the model [48]. At the same time, the high_metabolism features also helped facilitate the stratification of the mutations. Similar to the work in this study, Mu et al. selected the main habitat-related features for a model of the prognostic assessment of locally advanced cervical cancer based on ^18^F-FDG PET/CT images from the hypermetabolic regions [27]. Moreover, Wu et al. showed that the volume of most metabolically active and metabolically heterogeneous tumor solid components can be used as a predictor of overall survival and out-of-field progression in patients of lung cancer [49]. Based on this result, we hypothesize that the hypermetabolic habitats of tumors in PET images, which generally reflect the metabolism of active tumor cells, may harbor more information about mutations in tumors than hypometabolic habitat. It is evident from the results, that using 40% of SUVmax as the threshold to generate hypermetabolic VOI is a more convenient method to extract radiomics features from hypermetabolic tumor regions. Therefore, to further validate the effectiveness of the habitat-derived radiomic analysis, we generated a VOI with the SUVmax of 40% as the threshold based on the VOI of the whole tumor region obtained in this study, and carried out the same feature screening and modeling process. However, the model was severely overfitted during training (not shown in the results). Tumor heterogeneity refers not only to the heterogeneity within a tumor, but also to the heterogeneity between tumors. Therefore, using a fixed threshold to evaluate the degree of metabolism of all tumors interferes with the quantification of tumor heterogeneity based on radiomics features. Moreover, all the features considered here were wavelet features, which indicates the importance of wavelet decomposition for radiomics analysis. The wavelet features characterize heterogeneity at multiple spatial scales, and may be an important radiomic feature to quantify KRAS/NRAS/BRAF mutations [32].

Our analysis of clinicopathological factors showed that patients with high CEA levels and high TNM stages had a higher probability of KRAS/NRAS/BRAF mutations. KRAS/NRAS/BRAF mutations are associated with poor prognosis of patients with CRC [50, 51], while TNM staging prediction systems and markers such as CEA play an important role in monitoring the prognosis of gastrointestinal cancer [52, 53]. Therefore, there are potential associations between KRAS/NRAS/BRAF mutations and CEA level as well as TNM stage.

Deep learning has exhibited significant value in oncology [54, 55]. In particular, Wu et al. have reported that adding deep learning features to manual radiomic features can improve the ability to predict the KRAS mutation in case of CRC [56]. However, because of the small sample size used in this study, and given that deep learning is a data-intensive technique, we did not use it here. Its appropriate use in this domain is another important area that warrants further research.

This study has certain limitations. First, this was a preliminary exploration of a small sample collected from a single hospital. Our model delivered good predictive performance, and the results of its learning curve showed that it achieved convergence even with a small number of samples. However, using a larger sample size and providing external validation will further verify its clinical significance. Second, even though it is the mainstream method in radiomics research, manual segmentation may introduce uncertainty due to human participation. Automatic segmentation can further optimize research on the radiomics-based prediction of mutations in the CRC gene. Finally, the probability of KRAS/NRAS/BRAF mutations obtained in this study was higher than that reported in the literature, and the impact of this outcome on our model needs to be further evaluated.

## Conclusion

In this study, we showed that habitat-derived radiomic features are superior to conventional metabolic parameters and whole tumor region radiomic features in predicting KRAS/NRAS/BRAF mutations in CRC patients. In addition, the SVM model based on habitat-derived radiomic features has the potential to assist in decision-making regarding the treatment of CRC patients in clinical practice, but its performance needs to be validated on a larger prospective cohort than was considered here.

### Supplementary Information

**Additional file 1: Fig. S1. **Cluster heatmaps of habitat-derived radiomic features (a), whole-tumor radiomic features (b), and metabolic parameters (c). The X-axis represents 62 patients, and the Y-axis represents image features. Patients in the same cluster shared similar image features in Euclidean space. The semantic labels (mutant and wild types) for each patient are shown on the red and blue bars above. **Fig. S2.** (a) Heatmap of the correlation between habitat-derived radiomic features and whole-tumor radiomic features. (b) Heatmap of the correlation between habitat-derived radiomic features and metabolic parameters. **Fig. S3.** Feature selection of whole tumor region radiomics features using the LASSO algorithm. (a) Tuning parameter lambda selection in the LASSO algorithm used 10-fold cross-validation. (b) LASSO coefficient profiles of the features. LASSO, least absolute shrinkage and selection operator. **Fig. S4.** Violin plot of the distribution of the metabolic parameters. SUVmin, minimal standardized uptake value; SUVmean, average standardized uptake value; SUVmax, maximal standardized uptake value; SUVstd, standard deviation of standardized uptake value; MTV, metabolic tumor volume; sMTV, standardized metabolic tumor volume; TLG, total lesion glycolysis, sTLG, standardized total lesion glycolysis.

## Data Availability

The datasets used and/or analysed during the current study are available from the corresponding author on reasonable request.

## Abbreviations

AUC: Area under the receiver operating characteristic curve
AUPR: Area under the precision–recall curve
BRAF: V-raf murine sarcoma viral oncogene homolog B
CRC: Colorectal cancer
CT: Computed tomography
CEA: Carcinoembryonic antigen
DCA: Decision curve analysis
EGFR: Epidermal growth factor receptor
FDG: Fluorodeoxyglucose
glcm: Gray-level co-occurrence matrix
glszm: Gray-level size zone matrix
glrlm: Gray-level run length matrix
gldm: Gray-level dependence matrix
ICCs: Inter-observer and intra-observer correlation coefficients
KRAS: Kirsten rat sarcoma viral oncogene homolog
LASSO: Least absolute shrinkage and selection operator
MTV: Metabolic tumor volume
mRMR: Maximum relevance minimum redundancy
NRAS: Neuroblastoma rat sarcoma viral oncogene homolog
ngtdm: Neighboring gray tone difference matrix
PET: Positron emission tomography
Ras: Rat sarcoma viral oncogene
ROC: Receiver operating characteristic
SUV: Standardized uptake value
SUVmin: Minimal standardized uptake value
SUVmean: Average standardized uptake value
SUVmax: Maximal standardized uptake value
SUVstd: Standard deviation of standardized uptake value
sMTV: Standardized metabolic tumor volume
sTLG: Standardized total lesion glycolysis
SVM: Support vector machine
SHAP: SHapley Additive explanation
TLG: Total lesion glycolysis
VOI: Volume of interest

## References

[1] Qi J, Sun H, Zhang Y, Wang Z, Xun Z, Li Z (2022). Single-cell and spatial analysis reveal interaction of FAP+ fibroblasts and SPP1+ macrophages in colorectal cancer. Nat Commun.

[2] Wang TH, Wu CC, Huang KY, Chuang WY, Hsueh C, Li HJ (2020). Profiling of subcellular EGFR interactome reveals hnRNP A3 modulates nuclear EGFR localization. Oncogenesis.

[3] Denis JA, Patroni A, Guillerm E, Pépin D, Benali-Furet N, Wechsler J (2016). Droplet digital PCR of circulating tumor cells from colorectal cancer patients can predict KRAS mutations before surgery. Mol Oncol.

[4] He P, Zou Y, Qiu J, Yang T, Peng L, Zhang X (2021). Pretreatment ^18^F-FDG PET/CT imaging predicts the KRAS/NRAS/BRAF gene mutational status in colorectal cancer. J Oncol.

[5] Johnson H, El-Schich Z, Ali A, Zhang X, Simoulis A, Wingren AG (2022). Gene-mutation-based algorithm for prediction of treatment response in colorectal cancer patients. Cancers (Basel).

[6] Nicolazzo C, Belardinilli F, Vestri A, Magri V, De Renzi G, De Meo M (2022). *RAS* mutation conversion in bevacizumab-treated metastatic colorectal cancer patients: a liquid biopsy based study. Cancers (Basel).

[7] Madej E, Brożyna AA, Adamczyk A, Wronski N, Harazin-Lechowska A, Muzyk A (2023). Vemurafenib and dabrafenib downregulates RIPK4 level. Cancers (Basel).

[8] Liu J, Zeng W, Huang C, Wang J, Xu L, Ma D (2018). Upregulation of c-mesenchymal epithelial transition expression and *RAS* mutations are associated with late lung metastasis and poor prognosis in colorectal carcinoma. Exp Ther Med.

[9] Benson AB, Venook AP, Al-Hawary MM, Arain MA, Chen YJ, Ciombor KK (2021). Colon cancer, version 2.2021, NCCN clinical practice guidelines in oncology. J Natl Compr Canc Netw.

[10] Montagut C, Argilés G, Ciardiello F, Poulsen TT, Dienstmann R, Kragh M (2018). Efficacy of Sym004 in patients with metastatic colorectal cancer with acquired resistance to Anti-EGFR therapy and molecularly selected by circulating tumor DNA analyses: a phase 2 randomized clinical trial. JAMA Oncol.

[11] Morano F, Corallo S, Lonardi S, Raimondi A, Cremolini C, Rimassa L (2019). Negative hyperselection of patients with *RAS* and *BRAF* wild-type metastatic colorectal cancer who received panitumumab-based maintenance therapy. J Clin Oncol.

[12] Iwamoto M, Kawada K, Nakamoto Y, Itatani Y, Inamoto S, Toda K (2014). Regulation of 18F-FDG accumulation in colorectal cancer cells with mutated KRAS. J Nucl Med.

[13] Krikelis D, Skoura E, Kotoula V, Rondogianni P, Pianou N, Samartzis A (2014). Lack of association between KRAS mutations and 18F-FDG PET/CT in Caucasian metastatic colorectal cancer patients. Anticancer Res.

[14] Jiang P, Du W, Wang X, Mancuso A, Gao X, Wu M (2011). p53 regulates biosynthesis through direct inactivation of glucose-6-phosphate dehydrogenase. Nat Cell Biol.

[15] Taguchi N, Oda S, Yokota Y, Yamamura S, Imuta M, Tsuchigame T (2019). CT texture analysis for the prediction of KRAS mutation status in colorectal cancer via a machine learning approach. Eur J Radiol.

[16] Zinn PO, Singh SK, Kotrotsou A, Abrol S, Thomas G, Mosley J (2017). Distinct radiomic phenotypes define glioblastoma TP53-PTEN-EGFR mutational landscape. Neurosurg.

[17] Lovinfosse P, Koopmansch B, Lambert F, Jodogne S, Kustermans G, Hatt M (2016). (18)F-FDG PET/CT imaging in rectal cancer: relationship with the RAS mutational status. Br J Radiol.

[18] Cui Y, Liu H, Ren J, Du X, Xin L, Li D (2020). Development and validation of a MRI-based radiomics signature for prediction of KRAS mutation in rectal cancer. Eur Radiol.

[19] Meng X, Xia W, Xie P, Zhang R, Li W, Wang M (2019). Preoperative radiomic signature based on multiparametric magnetic resonance imaging for noninvasive evaluation of biological characteristics in rectal cancer. Eur Radiol.

[20] Gatenby RA, Grove O, Gillies RJ (2013). Quantitative imaging in cancer evolution and ecology. Radiology.

[21] Kim M, Park JE, Kim HS, Kim N, Park SY, Kim YH (2021). Spatiotemporal habitats from multiparametric physiologic MRI distinguish tumor progression from treatment-related change in post-treatment glioblastoma. Eur Radiol.

[22] Wu J, Cao G, Sun X, Lee J, Rubin DL, Napel S (2018). Intratumoral spatial heterogeneity at perfusion MR imaging predicts recurrence-free survival in locally advanced breast cancer treated with neoadjuvant chemotherapy. Radiology.

[23] Xie C, Yang P, Zhang X, Xu L, Wang X, Li X (2019). Sub-region based radiomics analysis for survival prediction in oesophageal tumours treated by definitive concurrent chemoradiotherapy. EBioMedicine.

[24] Chen SW, Shen WC, Chen WT, Hsieh TC, Yen KY, Chang JG (2019). Metabolic imaging phenotype using radiomics of [^18^F]FDG PET/CT associated with genetic alterations of colorectal cancer. Mol Imaging Biol.

[25] Nioche C, Orlhac F, Boughdad S, Reuzé S, Goya-Outi J, Robert C (2018). LIFEx: a freeware for radiomic feature calculation in multimodality imaging to accelerate advances in the characterization of tumor heterogeneity. Cancer Res.

[26] Wang X, Xu C, Grzegorzek M, Sun H (2022). Habitat radiomics analysis of pet/ct imaging in high-grade serous ovarian cancer: application to Ki-67 status and progression-free survival. Front Physiol.

[27] Mu W, Liang Y, Hall LO, Tan Y, Balagurunathan Y, Wenham R (2020). ^18^F-FDG PET/CT habitat radiomics predicts outcome of patients with cervical cancer treated with chemoradiotherapy. Radiol Artif Intell.

[28] van Griethuysen JJM, Fedorov A, Parmar C, Hosny A, Aucoin N, Narayan V (2017). Computational radiomics system to decode the radiographic phenotype. Cancer Res.

[29] Pfaehler E, Mesotten L, Zhovannik I, Pieplenbosch S, Thomeer M, Vanhove K (2021). Plausibility and redundancy analysis to select FDG-PET textural features in non-small cell lung cancer. Med Phys.

[30] Somasundaram A, García DV, Pfaehler E, Jauw YWS, Zijlstra JM, van Dongen GAMS (2022). Noise sensitivity of ^89^Zr-Immuno-PET radiomics based on count-reduced clinical images. EJNMMI Phys.

[31] Wang J, Xiong X, Ye J, Yang Y, He J, Liu J (2022). A radiomics nomogram for classifying hematoma entities in acute spontaneous intracerebral hemorrhage on non-contrast-enhanced computed tomography. Front Neurosci.

[32] Wang X, Zhao X, Li Q, Xia W, Peng Z, Zhang R (2019). Can peritumoral radiomics increase the efficiency of the prediction for lymph node metastasis in clinical stage T1 lung adenocarcinoma on CT?. Eur Radiol.

[33] Uttam S, Stern AM, Sevinsky CJ, Furman S, Pullara F, Spagnolo D (2020). Spatial domain analysis predicts risk of colorectal cancer recurrence and infers associated tumor microenvironment networks. Nat Commun.

[34] Cheng CW, Zhou Y, Pan WH, Dey S, Wu CY, Hsu WL (2018). Hierarchical and programmable one-pot synthesis of oligosaccharides. Nat Commun.

[35] Zhao H, Su Y, Wang M, Lyu Z, Xu P, Jiao Y (2022). The machine learning model for distinguishing pathological subtypes of non-small cell lung cancer. Front Oncol.

[36] Chai KE, Anthony S, Coiera E, Magrabi F (2013). Using statistical text classification to identify health information technology incidents. J Am Med Inform Assoc.

[37] Choi HJ, Kim I, Lee HJ, Oh HJ, Ahn MK, Baek WI (2022). Clinical characteristics of neonatal cholestasis in a tertiary hospital and the development of a novel prediction model for mortality. EBioMedicine.

[38] Yang L, Dong D, Fang M, Zhu Y, Zang Y, Liu Z (2018). Can CT-based radiomics signature predict KRAS/NRAS/BRAF mutations in colorectal cancer?. Eur Radiol.

[39] Srivatsa S, Paul MC, Cardone C, Holcmann M, Amberg N, Pathria P (2017). EGFR in tumor-associated myeloid cells promotes development of colorectal cancer in mice and associates with outcomes of patients. Gastroenterology.

[40] Sclafani F, Chau I, Cunningham D, Hahne JC, Vlachogiannis G, Eltahir Z (2018). KRAS and BRAF mutations in circulating tumour DNA from locally advanced rectal cancer. Sci Rep.

[41] Liu X, Wang SC, Ni M, Xie Q, Zhang YF, Lv WF (2022). Correlation between ^18^F-FDG PET/CT intra-tumor metabolic heterogeneity parameters and KRAS mutation in colorectal cancer. Abdom Radiol (NY).

[42] Cho HH, Kim H, Nam SY, Lee JE, Han BK, Ko EY (2022). Measurement of perfusion heterogeneity within tumor habitats on magnetic resonance imaging and its association with prognosis in breast cancer patients. Cancers (Basel).

[43] Gou W, Ling CW, He Y, Jiang Z, Fu Y, Xu F (2021). Interpretable machine learning framework reveals robust gut microbiome features associated with type 2 diabetes. Diabetes Care.

[44] Liu CL, Tain YL, Lin YC, Hsu CN (2022). Prediction and clinically important factors of acute kidney injury non-recovery. Front Med (Lausanne).

[45] Cui L, Yu T, Kan Y, Dong Y, Luo Y, Jiang X (2022). Multi-parametric MRI-based peritumoral radiomics on prediction of lymph-vascular space invasion in early-stage cervical cancer. Diagn Interv Radiol.

[46] Zhang G, Chen L, Liu A, Pan X, Shu J, Han Y (2021). Comparable performance of deep learning-based to manual-based tumor segmentation in KRAS/NRAS/BRAF mutation prediction with MR-based radiomics in rectal cancer. Front Oncol.

[47] Tang X, Huang H, Du P, Wang L, Yin H, Xu X (2022). Intratumoral and peritumoral CT-based radiomics strategy reveals distinct subtypes of non-small-cell lung cancer. J Cancer Res Clin Oncol.

[48] He K, Liu X, Li M, Li X, Yang H, Zhang H (2020). Noninvasive KRAS mutation estimation in colorectal cancer using a deep learning method based on CT imaging. BMC Med Imaging.

[49] Wu J, Gensheimer MF, Dong X, Rubin DL, Napel S, Diehn M (2016). Robust intratumor partitioning to identify high-risk subregions in lung cancer: a pilot study. Int J Radiat Oncol Biol Phys.

[50] Smeby J, Sveen A, Merok MA, Danielsen SA, Eilertsen IA, Guren MG (2018). CMS-dependent prognostic impact of KRAS and BRAFV600E mutations in primary colorectal cancer. Ann Oncol.

[51] Yanai Y, Hayashi T, Akazawa Y, Yatagai N, Tsuyama S, Yao T (2020). Clinicopathological and mutational differences between tumors with multiple metastases and single lung metastasis in colorectal cancer. Oncol Lett.

[52] Peng Q, Zhao P, Shen Y, Cheng M, Wu Y, Zhu Y (2020). Prognostic implication and functional exploration for microRNA-20a as a molecular biomarker of gastrointestinal cancer. BMC Cancer.

[53] Thirunavukarasu P, Talati C, Munjal S, Attwood K, Edge SB, Francescutti V (2015). Effect of incorporation of pretreatment serum carcinoembryonic antigen levels into AJCC staging for colon cancer on 5-year survival. JAMA Surg.

[54] Zhao H, Su Y, Lyu Z, Tian L, Xu P, Lin L (2023). Non-invasively discriminating the pathological subtypes of non-small cell lung cancer with pretreatment ^18^F-FDG PET/CT using deep learning. Acad Radiol.

[55] Wang S, Yu H, Gan Y, Wu Z, Li E, Li X (2022). Mining whole-lung information by artificial intelligence for predicting EGFR genotype and targeted therapy response in lung cancer: a multicohort study. Lancet Digit Health.

[56] Wu X, Li Y, Chen X, Huang Y, He L, Zhao K (2020). Deep learning features improve the performance of a radiomics signature for predicting KRAS status in patients with colorectal cancer. Acad Radiol.

