# Supplementary material for: Using tumor habitat-derived radiomic analysis during pretreatment 18F-FDG PET for predicting KRAS/NRAS/BRAF mutations in colorectal cancer
Source: Cancer Imaging. 2024 Feb 12;24:26. doi: 10.1186/s40644-024-00670-2 (PMC10860234; doi:10.1186/s40644-024-00670-2)
Supplement: Supplementary file 1 — Additional file 1: Fig. S1. Cluster heatmaps of habitat-derived radiomic features (a), whole-tumor radiomic features (b), and metabolic parameters (c). The X-axis represents 62 patients, and the Y-axis represents image features. Patients in the same cluster shared similar image features in Euclidean space. The semantic labels (mutant and wild types) for each patient are shown on the red and blue bars above. Fig. S2. (a) Heatmap of the correlation between habitat-derived radiomic features and whole-tumor radiomic features. (b) Heatmap of the correlation between habitat-derived radiomic features and metabolic parameters. Fig. S3. Feature selection of whole tumor region radiomics features using the LASSO algorithm. (a) Tuning parameter lambda selection in the LASSO algorithm used 10-fold cross-validation. (b) LASSO coefficient profiles of the features. LASSO, least absolute shrinkage and selection operator. Fig. S4. Violin plot of the distribution of the metabolic parameters. SUVmin, minimal standardized uptake value; SUVmean, average standardized uptake value; SUVmax, maximal standardized uptake value; SUVstd, standard deviation of standardized uptake value; MTV, metabolic tumor volume; sMTV, standardized metabolic tumor volume; TLG, total lesion glycolysis, sTLG, standardized total lesion glycolysis. [file 40644_2024_670_MOESM1_ESM.docx]

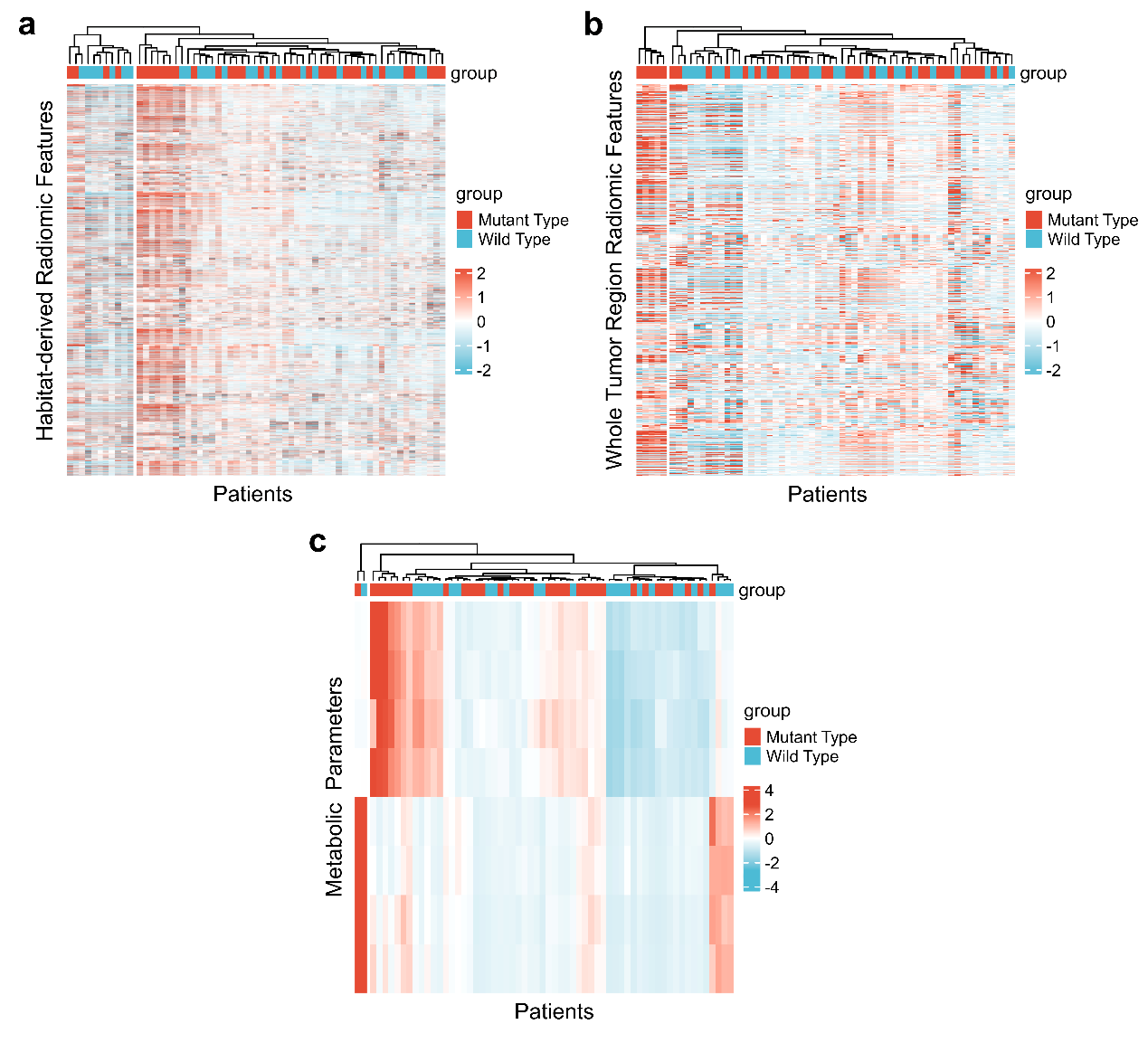
**Supplementary Information**

**Fig.S1** Cluster heatmaps of habitat-derived radiomic features (a), whole-tumor radiomic features (b), and metabolic parameters (c). The X-axis represents 62 patients, and the Y-axis represents image features. Patients in the same cluster shared similar image features in Euclidean space. The semantic labels (mutant and wild types) for each patient are shown on the red and blue bars above.


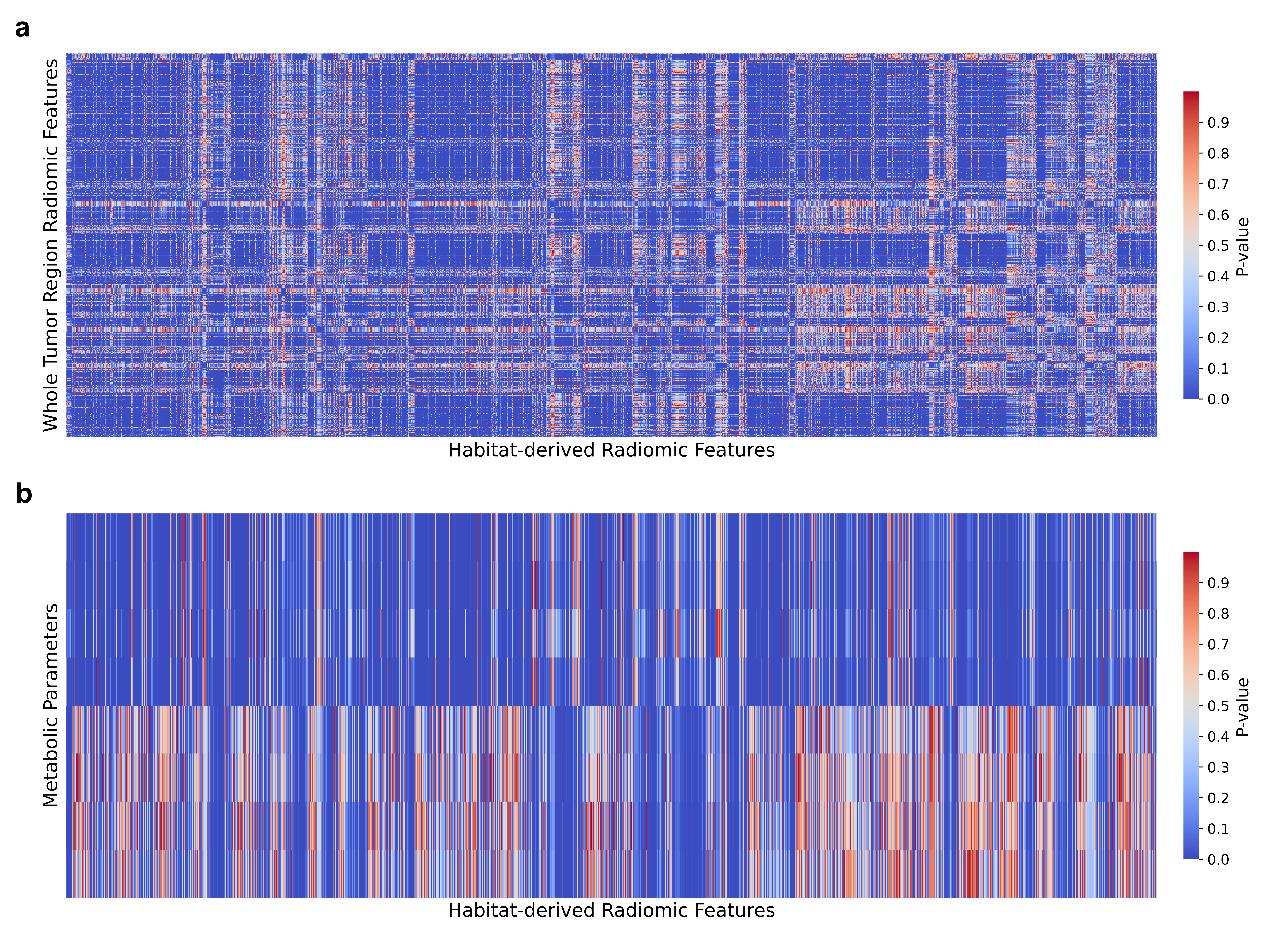


**Fig.S2** (a) Heatmap of the correlation between habitat-derived radiomic features and whole-tumor radiomic features. (b) Heatmap of the correlation between habitat-derived radiomic features and metabolic parameters.

**
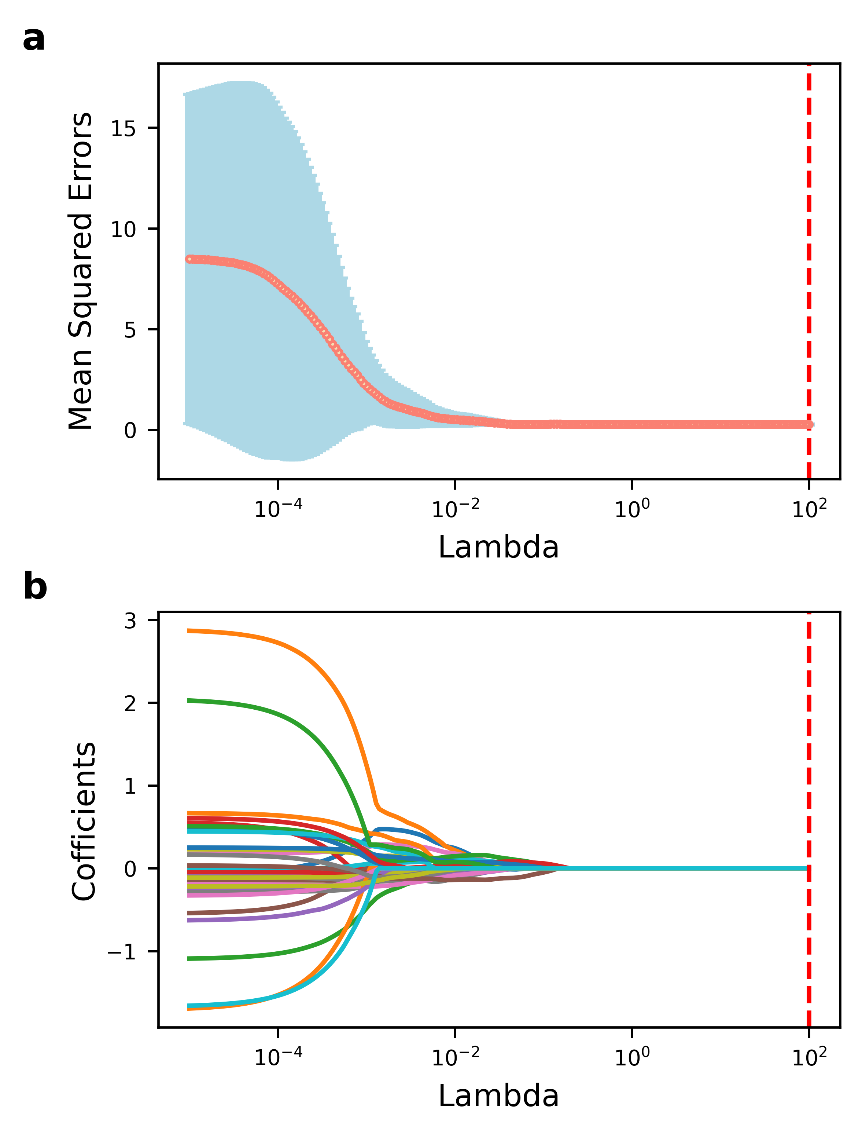
Fig.S3** Feature selection of whole tumor region radiomics features using the LASSO algorithm. (a) Tuning parameter lambda selection in the LASSO algorithm used 10-fold cross-validation. (b) LASSO coefficient profiles of the features. LASSO, least absolute shrinkage and selection operator.


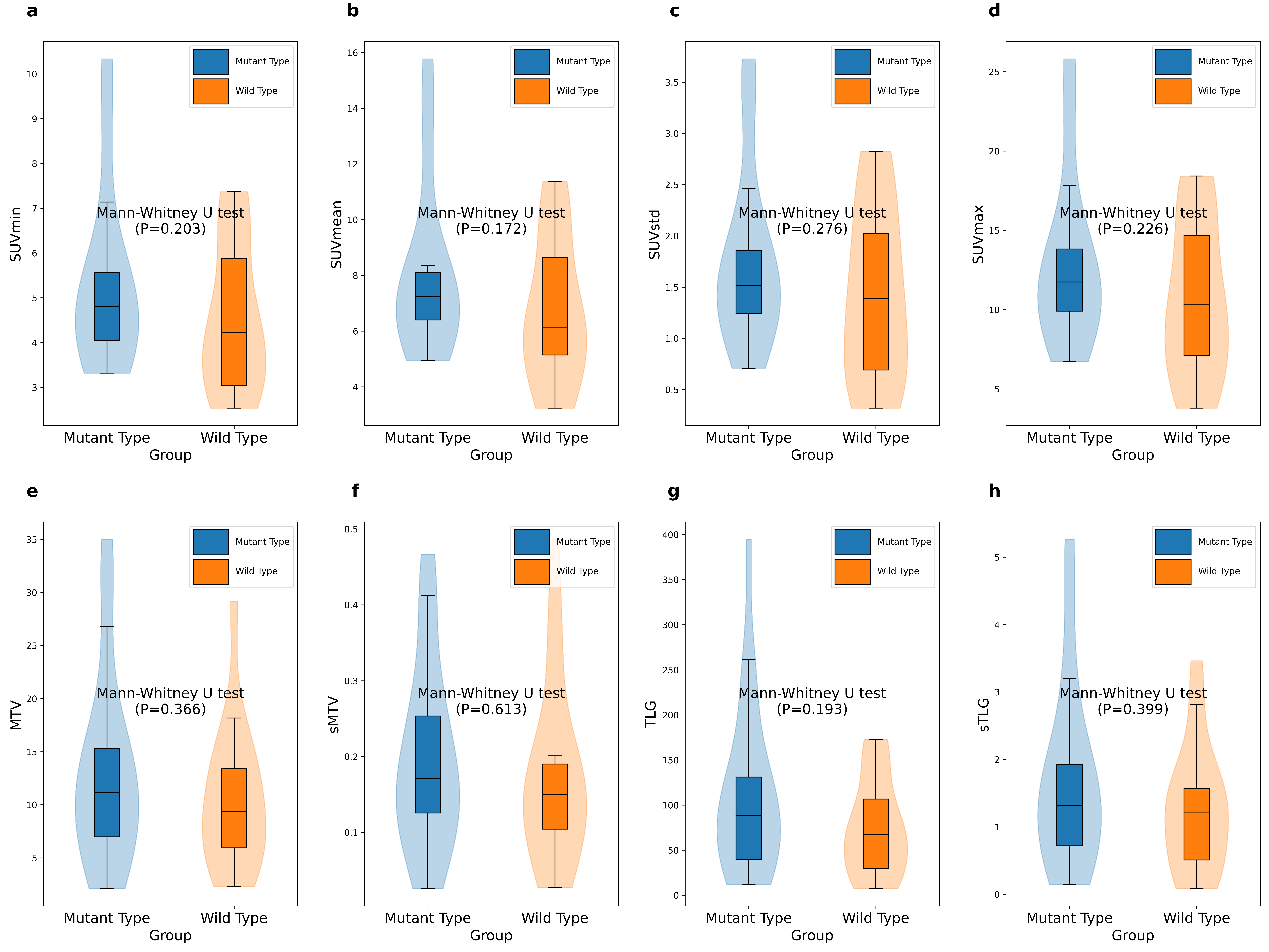
**Fig.S4** Violin plot of the distribution of the metabolic parameters. SUVmin, minimal standardized uptake value; SUVmean, average standardized uptake value; SUVmax, maximal standardized uptake value; SUVstd, standard deviation of standardized uptake value; MTV, metabolic tumor volume; sMTV, standardized metabolic tumor volume; TLG, total lesion glycolysis, sTLG, standardized total lesion glycolysis
